# Supplementary material for: Mechanisms governing the pioneering and redistribution capabilities of the non-classical pioneer PU.1
Source: Nat Commun. 2020 Jan 21;11:402. doi: 10.1038/s41467-019-13960-2 (PMC6972792; doi:10.1038/s41467-019-13960-2)
Supplement: Supplementary file 7 — Source data [file 41467_2019_13960_MOESM7_ESM.zip › Source_Data/FigureS2/FigureS2B_MotifScanOutput/KmeansCluster8/homerResults.html]

/misc/data/analysis/project\_PU1/CTV1/motifs/basic/PU1\_Kmeans14.8/final - Homer de novo Motif Results


# Homer *de novo* Motif Results (/misc/data/analysis/project\_PU1/CTV1/motifs/basic/PU1\_Kmeans14.8/final)

Known Motif Enrichment Results  
Gene Ontology Enrichment Results  
If Homer is having trouble matching a motif to a known motif, try copy/pasting the matrix file into
STAMP  
More information on motif finding results: HOMER
| Description of Results
| Tips
  
Total target sequences = 1512  
Total background sequences = 45416  
\* - possible false positive  

|  |  |  |  |  |  |  |  |  |
| --- | --- | --- | --- | --- | --- | --- | --- | --- |
| Rank | Motif | P-value | log P-pvalue | % of Targets | % of Background | STD(Bg STD) | Best Match/Details | Motif File |
| 1 | G A T C C T G A A T G C G C A T G A C T A G T C A G T C A G C T A G T C G C A T G C A T G A C T | 1e-869 | -2.002e+03 | 79.03% | 8.97% | 39.4bp (76.9bp) | PB0058.1\_Sfpi1\_1/Jaspar(0.965) More Information \| Similar Motifs Found | motif file (matrix) |
| 2 | T C A G C T G A T C G A T A G C G T C A C T G A T C A G C A T G T G C A C G T A A C T G C G A T A C T G T C A G | 1e-103 | -2.375e+02 | 15.34% | 2.37% | 48.2bp (77.8bp) | ELF5(ETS)/T47D-ELF5-ChIP-Seq(GSE30407)/Homer(0.796) More Information \| Similar Motifs Found | motif file (matrix) |
| 3 | C T G A A T G C G C A T A C G T A G T C G A C T T A C G G A T C G A C T C G A T G A C T A G C T G A C T | 1e-90 | -2.083e+02 | 11.84% | 1.51% | 43.9bp (76.6bp) | SPI1/MA0080.4/Jaspar(0.797) More Information \| Similar Motifs Found | motif file (matrix) |
| 4 | C T A G A G C T A C G T A G C T A G T C A G T C A C G T | 1e-88 | -2.035e+02 | 44.58% | 21.11% | 55.9bp (74.5bp) | EWS:ERG-fusion(ETS)/CADO\_ES1-EWS:ERG-ChIP-Seq(SRA014231)/Homer(0.814) More Information \| Similar Motifs Found | motif file (matrix) |
| 5 | G A T C G C T A T C G A A G T C G T A C C G T A A T G C G C T A C A G T T A G C | 1e-85 | -1.975e+02 | 27.78% | 9.53% | 53.3bp (69.0bp) | RUNX1(Runt)/Jurkat-RUNX1-ChIP-Seq(GSE29180)/Homer(0.909) More Information \| Similar Motifs Found | motif file (matrix) |
| 6 | A G C T A G C T C T G A A G T C C G A T C G A T A G T C A G T C A G T C A C G T G C T A | 1e-79 | -1.838e+02 | 12.10% | 1.89% | 48.9bp (69.7bp) | HNRNPA2B1(RRM)/Homo\_sapiens-RNCMPT00024-PBM/HughesRNA(0.757) More Information \| Similar Motifs Found | motif file (matrix) |
| 7 | C G T A C T G A G T A C C G T A A T C G C T G A A C T G C G T A C G T A A T G C A C G T | 1e-65 | -1.498e+02 | 10.78% | 1.88% | 52.2bp (71.1bp) | SRSF10(RRM)/Homo\_sapiens-RNCMPT00019-PBM/HughesRNA(0.718) More Information \| Similar Motifs Found | motif file (matrix) |
| 8 | T C G A A G T C G C A T G A C T G A T C G T A C G A C T G T A C A C G T A T C G G A T C | 1e-58 | -1.353e+02 | 17.13% | 5.24% | 57.3bp (73.0bp) | PU.1(ETS)/ThioMac-PU.1-ChIP-Seq(GSE21512)/Homer(0.757) More Information \| Similar Motifs Found | motif file (matrix) |
| 9 | T C G A C T G A A T G C C A G T A C G T A G T C A G T C G A C T A G T C C G T A T C A G C T G A T C A G | 1e-44 | -1.025e+02 | 10.19% | 2.49% | 50.2bp (74.3bp) | STAT6(Stat)/Macrophage-Stat6-ChIP-Seq(GSE38377)/Homer(0.786) More Information \| Similar Motifs Found | motif file (matrix) |
| 10 | C T G A T A C G C G T A A T C G T A C G T G C A G C T A T A C G C T G A C A T G T C A G C T G A C G T A T C A G | 1e-41 | -9.569e+01 | 6.68% | 1.11% | 42.1bp (87.1bp) | Trl/dmmpmm(SeSiMCMC)/fly(0.690) More Information \| Similar Motifs Found | motif file (matrix) |
| 11 | G T C A T G A C C G T A A T C G G T C A C A T G A T C G T G C A C G T A A T C G C G A T A T C G T G C A G T C A | 1e-39 | -9.160e+01 | 6.94% | 1.28% | 44.8bp (71.8bp) | PU.1(ETS)/ThioMac-PU.1-ChIP-Seq(GSE21512)/Homer(0.752) More Information \| Similar Motifs Found | motif file (matrix) |
| 12 | C G T A T C G A A C T G A T C G A C G T T C A G C G T A G A T C A C T G C G A T C T G A | 1e-38 | -8.804e+01 | 5.03% | 0.64% | 52.0bp (71.4bp) | SPIC/MA0687.1/Jaspar(0.700) More Information \| Similar Motifs Found | motif file (matrix) |
| 13 | A C T G A G T C A C G T A C G T A G T C A G T C A G T C | 1e-33 | -7.782e+01 | 14.55% | 5.73% | 59.4bp (72.9bp) | GCR2(MacIsaac)/Yeast(0.915) More Information \| Similar Motifs Found | motif file (matrix) |
| 14 | C G T A A C T G A T G C C G T A A C T G C G T A C G A T | 1e-32 | -7.396e+01 | 22.16% | 11.19% | 56.7bp (70.6bp) | Tal1(0.790) More Information \| Similar Motifs Found | motif file (matrix) |
| 15 | A C G T A C G T A G T C A G T C G C A T A G C T A G T C A G T C A C G T C T A G G A C T A G C T A G C T A C G T | 1e-29 | -6.750e+01 | 6.55% | 1.56% | 46.4bp (73.9bp) | RBM5(Znf)/Homo\_sapiens-RNCMPT00055-PBM/HughesRNA(0.666) More Information \| Similar Motifs Found | motif file (matrix) |
| 16 | A C T G C G T A A C T G C G T A C G T A A T C G A C G T G C A T | 1e-29 | -6.748e+01 | 16.27% | 7.35% | 49.1bp (76.0bp) | RNP4F(RRM)/Drosophila\_melanogaster-RNCMPT00060-PBM/HughesRNA(0.778) More Information \| Similar Motifs Found | motif file (matrix) |
| 17 | A G T C C G T A A C T G A C G T C G A T A G T C A G C T A C T G A G T C A G C T A G C T G T A C A G C T | 1e-29 | -6.717e+01 | 6.15% | 1.39% | 45.9bp (70.5bp) | SpiB(ETS)/OCILY3-SPIB-ChIP-Seq(GSE56857)/Homer(0.633) More Information \| Similar Motifs Found | motif file (matrix) |
| 18 | A C T G C T A G T C G A C T G A T C A G G C A T A C T G A G T C G A T C T G C A A T G C T G C A | 1e-24 | -5.732e+01 | 3.37% | 0.45% | 45.7bp (75.2bp) | PU.1-IRF(ETS:IRF)/Bcell-PU.1-ChIP-Seq(GSE21512)/Homer(0.686) More Information \| Similar Motifs Found | motif file (matrix) |
| 19 | C G A T A C T G C T G A A C T G A C T G C G T A C G T A A T G C C G T A A C T G G T A C | 1e-24 | -5.729e+01 | 4.30% | 0.79% | 52.8bp (81.5bp) | ZNF189(Zf)/HEK293-ZNF189.GFP-ChIP-Seq(GSE58341)/Homer(0.791) More Information \| Similar Motifs Found | motif file (matrix) |
| 20 | A T C G A T C G G T A C C G T A T A G C A G T C G A T C G T C A G T A C T C G A | 1e-24 | -5.705e+01 | 5.16% | 1.15% | 53.3bp (71.3bp) | RUNX2(Runt)/PCa-RUNX2-ChIP-Seq(GSE33889)/Homer(0.767) More Information \| Similar Motifs Found | motif file (matrix) |
| 21 | C A G T A G T C G C A T A C G T A G T C G A C T T G A C A G T C C T G A C A G T G C A T A C G T T G A C | 1e-24 | -5.530e+01 | 5.09% | 1.16% | 54.6bp (67.6bp) | br-Z2/dmmpmm(Bergman)/fly(0.774) More Information \| Similar Motifs Found | motif file (matrix) |
| 22 | A C T G A C G T C T A G A C T G A C T G G T A C A C T G C T A G A G C T | 1e-23 | -5.306e+01 | 14.48% | 6.92% | 60.6bp (67.9bp) | Egr2(Zf)/Thymocytes-Egr2-ChIP-Seq(GSE34254)/Homer(0.863) More Information \| Similar Motifs Found | motif file (matrix) |
| 23 | C T A G C T A G C A T G C T A G G T C A C T G A T C A G T A G C C G T A T A C G A T G C T A G C C G T A A T C G | 1e-22 | -5.216e+01 | 2.51% | 0.25% | 48.6bp (79.2bp) | RBPJ:Ebox(?,bHLH)/Panc1-Rbpj1-ChIP-Seq(GSE47459)/Homer(0.669) More Information \| Similar Motifs Found | motif file (matrix) |
| 24 | C T G A T G C A G T C A T C G A C A T G A C T G A C T G C T A G T C G A C T G A A C T G A T G C G C T A | 1e-20 | -4.776e+01 | 2.38% | 0.25% | 61.3bp (74.8bp) | SPIC/MA0687.1/Jaspar(0.758) More Information \| Similar Motifs Found | motif file (matrix) |
| 25 | T C G A C A G T C G T A C T G A A G T C C A G T T G A C G C T A T A G C A C G T | 1e-20 | -4.746e+01 | 3.17% | 0.51% | 54.7bp (66.7bp) | MYB.PH3(2)(MYB)/Petunia hybrida/AthaMap(0.746) More Information \| Similar Motifs Found | motif file (matrix) |
| 26 | C T G A A C G T C A G T C A G T G T A C G T A C A G C T A G T C G T C A C G A T G A T C A C G T | 1e-19 | -4.497e+01 | 3.84% | 0.81% | 47.6bp (70.0bp) | JUN/MA0488.1/Jaspar(0.725) More Information \| Similar Motifs Found | motif file (matrix) |
| 27 | A C G T A G T C G A C T G A T C T C G A C G A T C T A G C T G A T A C G T C G A C G T A A T G C C A G T T A G C | 1e-19 | -4.428e+01 | 3.84% | 0.83% | 45.1bp (80.4bp) | GFX(?)/Promoter/Homer(0.677) More Information \| Similar Motifs Found | motif file (matrix) |
| 28 | C G A T A T G C C G A T C G T A A C T G A G T C C G T A A G T C G C A T A C G T A G T C A G T C | 1e-19 | -4.420e+01 | 1.72% | 0.11% | 46.1bp (37.0bp) | byn/dmmpmm(Bigfoot)/fly(0.675) More Information \| Similar Motifs Found | motif file (matrix) |
| 29 | A G T C A C G T C G T A A G C T A G T C G T C A A G C T T C A G C G T A A C T G G T C A C T G A A G T C C G T A | 1e-19 | -4.415e+01 | 2.45% | 0.31% | 57.7bp (72.4bp) | FZF1/MA0298.1/Jaspar(0.657) More Information \| Similar Motifs Found | motif file (matrix) |
| 30 | C A T G T C G A A C G T C A T G C G T A A T G C A C G T G T A C C T G A | 1e-19 | -4.377e+01 | 15.94% | 8.58% | 57.1bp (69.3bp) | Fra1(bZIP)/BT549-Fra1-ChIP-Seq(GSE46166)/Homer(0.974) More Information \| Similar Motifs Found | motif file (matrix) |
| 31 | A C G T C G A T A C G T G C A T T G C A C T G A C T G A T A G C G T C A C T G A T G A C A G T C | 1e-18 | -4.318e+01 | 3.17% | 0.57% | 41.4bp (65.7bp) | TCX2(CPP)/colamp-TCX2-DAP-Seq(GSE60143)/Homer(0.667) More Information \| Similar Motifs Found | motif file (matrix) |
| 32 | A C G T T C A G T A G C G C A T C G A T A T G C A G C T C A T G A G T C | 1e-18 | -4.197e+01 | 10.78% | 4.98% | 53.8bp (70.7bp) | odd/dmmpmm(Noyes)/fly(0.700) More Information \| Similar Motifs Found | motif file (matrix) |
| 33 | C A G T G T A C C G T A A G T C A C G T A G T C C G T A A G C T G A C T C T A G A C G T | 1e-17 | -4.105e+01 | 3.17% | 0.61% | 54.9bp (76.7bp) | z/dmmpmm(Down)/fly(0.818) More Information \| Similar Motifs Found | motif file (matrix) |
| 34 | A G T C A G T C A G T C C T G A A C G T C T A G C G T A G A C T G A T C A G T C C G T A C G T A C G A T A G T C | 1e-17 | -4.047e+01 | 1.79% | 0.15% | 75.2bp (56.0bp) | AARE(HLH)/mES-cMyc-ChIP-Seq/Homer(0.711) More Information \| Similar Motifs Found | motif file (matrix) |
| 35 | A G T C G C T A A T G C C T G A A G T C A G C T A C G T A C G T C G A T T G C A C T G A T C G A G T A C T G C A | 1e-17 | -3.975e+01 | 1.65% | 0.13% | 38.6bp (83.3bp) | CDF3(C2C2dof)/colamp-CDF3-DAP-Seq(GSE60143)/Homer(0.660) More Information \| Similar Motifs Found | motif file (matrix) |
| 36 | A T G C C A G T G T C A A G C T G A T C G C T A A G C T C T A G C T A G A C T G | 1e-17 | -3.937e+01 | 4.56% | 1.29% | 57.7bp (62.6bp) | FZF1/MA0298.1/Jaspar(0.731) More Information \| Similar Motifs Found | motif file (matrix) |
| 37 | A T C G A G T C G C T A A G T C G A T C A G C T A G T C G T A C A G T C G T C A A T G C G C T A A G C T G A T C | 1e-17 | -3.933e+01 | 1.52% | 0.10% | 52.5bp (60.6bp) | Sp5(Zf)/mES-Sp5.Flag-ChIP-Seq(GSE72989)/Homer(0.669) More Information \| Similar Motifs Found | motif file (matrix) |
| 38 | C T G A A C T G C G T A C G A T A G T C A C G T A C G T T C G A A C G T A T C G | 1e-16 | -3.894e+01 | 2.98% | 0.56% | 35.8bp (74.2bp) | GATA14(C2C2gata)/col-GATA14-DAP-Seq(GSE60143)/Homer(0.859) More Information \| Similar Motifs Found | motif file (matrix) |
| 39 | C G A T T A C G C G T A A C T G G T C A C G T A G A T C A C G T T A G C C G T A A T G C G A C T A G T C C G T A | 1e-16 | -3.813e+01 | 2.31% | 0.33% | 57.4bp (73.0bp) | PH0037.1\_Hdx/Jaspar(0.668) More Information \| Similar Motifs Found | motif file (matrix) |
| 40 | T G C A A T G C C G A T A G C T C G T A A G T C G C A T C G T A G C A T G A T C C G T A G A C T C T A G | 1e-16 | -3.759e+01 | 3.04% | 0.62% | 59.9bp (66.7bp) | FZF1/MA0298.1/Jaspar(0.620) More Information \| Similar Motifs Found | motif file (matrix) |
| 41 | G C A T T G C A G T A C C T A G A C G T A C G T A G C T A G T C A G T C G C A T T A C G C G T A A C T G A T C G | 1e-16 | -3.754e+01 | 2.98% | 0.59% | 67.0bp (70.9bp) | EWS:ERG-fusion(ETS)/CADO\_ES1-EWS:ERG-ChIP-Seq(SRA014231)/Homer(0.717) More Information \| Similar Motifs Found | motif file (matrix) |
| 42 | A G C T A C G T A G T C G A T C C G A T T C A G C G T A C T A G C T A G T A G C A G T C G C A T G A T C | 1e-15 | -3.461e+01 | 3.37% | 0.82% | 68.2bp (69.9bp) | TFAP2B(var.2)/MA0812.1/Jaspar(0.685) More Information \| Similar Motifs Found | motif file (matrix) |
| 43 | C A T G A C T G G A T C A C G T A C T G C G A T A G T C G A C T T G A C G T C A | 1e-15 | -3.460e+01 | 4.17% | 1.21% | 54.4bp (72.9bp) | Bach2(bZIP)/OCILy7-Bach2-ChIP-Seq(GSE44420)/Homer(0.726) More Information \| Similar Motifs Found | motif file (matrix) |
| 44 | A G T C C G A T C G A T G T A C A G T C A T G C A T G C C G A T A T G C A T G C | 1e-14 | -3.338e+01 | 7.47% | 3.23% | 54.1bp (75.3bp) | RO3G\_00049(RRM)/Rhizopus\_oryzae-RNCMPT00205-PBM/HughesRNA(0.778) More Information \| Similar Motifs Found | motif file (matrix) |
| 45 | A G C T C G T A A G T C C G T A A G T C C G T A A G T C C G A T A C G T | 1e-14 | -3.249e+01 | 4.70% | 1.57% | 52.0bp (74.2bp) | Rbm38(RRM)/Danio\_rerio-RNCMPT00283-PBM/HughesRNA(0.749) More Information \| Similar Motifs Found | motif file (matrix) |
| 46 | A T G C C G T A C T A G C G A T A C G T A T G C A G T C A C G T T A G C G C A T A C G T A C G T A G T C | 1e-14 | -3.247e+01 | 1.46% | 0.13% | 58.9bp (52.4bp) | SpiB(ETS)/OCILY3-SPIB-ChIP-Seq(GSE56857)/Homer(0.775) More Information \| Similar Motifs Found | motif file (matrix) |
| 47 | G C T A A C G T T A C G C G A T A C T G A C T G C A G T A T C G C T G A C T A G | 1e-14 | -3.239e+01 | 5.03% | 1.76% | 56.7bp (65.6bp) | BRUNOL6(RRM)/Homo\_sapiens-RNCMPT00187-PBM/HughesRNA(0.792) More Information \| Similar Motifs Found | motif file (matrix) |
| 48 | G T A C A G T C A G C T A G C T T G A C A G C T A C T G G A T C A G T C G T C A A G T C T A C G T G A C A C G T | 1e-12 | -2.989e+01 | 2.05% | 0.35% | 56.9bp (68.3bp) | achi/MA0207.1/Jaspar(0.633) More Information \| Similar Motifs Found | motif file (matrix) |
| 49 | T G C A A G T C G T C A C T G A T C A G A C G T A C G T G T A C G T A C A C G T C T A G T A G C A C G T A C G T | 1e-12 | -2.976e+01 | 0.79% | 0.02% | 47.1bp (22.5bp) | SPDEF(ETS)/VCaP-SPDEF-ChIP-Seq(SRA014231)/Homer(0.664) More Information \| Similar Motifs Found | motif file (matrix) |
| 50 | T C G A C A G T A G T C C G T A A G C T C T A G A C T G C T A G C T A G A T C G | 1e-12 | -2.969e+01 | 2.78% | 0.66% | 69.2bp (70.3bp) | Tv\_0259(RRM)/Trichomonas\_vaginalis-RNCMPT00259-PBM/HughesRNA(0.704) More Information \| Similar Motifs Found | motif file (matrix) |
| 51 | A C G T A C G T C G T A G T C A A G C T C T G A A C T G G T A C A C G T A C G T A T G C A C T G A C G T A C T G | 1e-12 | -2.881e+01 | 0.66% | 0.01% | 63.0bp (43.1bp) | Hmx3/MA0898.1/Jaspar(0.636) More Information \| Similar Motifs Found | motif file (matrix) |
| 52 | T A G C G T A C G A C T A G T C C G T A G A T C A C G T C G A T A G T C A G T C C T G A A G T C A G C T A G C T | 1e-12 | -2.872e+01 | 1.06% | 0.06% | 51.8bp (61.9bp) | SpiB(ETS)/OCILY3-SPIB-ChIP-Seq(GSE56857)/Homer(0.791) More Information \| Similar Motifs Found | motif file (matrix) |
| 53 | A C G T G C T A C T G A A T G C T C A G T C G A A G C T C G T A A C T G | 1e-12 | -2.811e+01 | 8.93% | 4.54% | 54.2bp (66.4bp) | AT3G25990(Trihelix)/colamp-AT3G25990-DAP-Seq(GSE60143)/Homer(0.749) More Information \| Similar Motifs Found | motif file (matrix) |
| 54 | A G T C G C T A A C G T A T G C A C G T A C T G G T A C A G T C G A C T G T A C C G T A | 1e-12 | -2.792e+01 | 7.08% | 3.26% | 58.0bp (70.7bp) | FOS::JUN/MA0099.2/Jaspar(0.668) More Information \| Similar Motifs Found | motif file (matrix) |
| 55 | A G T C A T C G C A G T C A G T A G T C G T A C C A G T T A C G G A T C | 1e-12 | -2.768e+01 | 10.25% | 5.53% | 56.5bp (73.4bp) | grh/dmmpmm(Papatsenko)/fly(0.744) More Information \| Similar Motifs Found | motif file (matrix) |
